# Medical Opinion and Movement

**Published:** 1910-05-28

**Authors:** 


### Medical Opinion and Movement.

### A New Theory of Anæsthesia.

Dr. Burker, of Tubingen, has carried out some interesting experiments with ether, from which he deduces a new theory to explain the narcotic effect of this and other anæsthetics. By adding ether to water in a voltameter he finds that a large quantity of gas is evolved at the cathode, composed of hydrogen and ether in definite proportions according to the law of Faraday. At the anode he obtained carbonic acid gas, carbon monoxide, and acetaldehyde. He suggests, therefore, that the anæsthetic action of these narcotics results from their exhausting the supply of oxygen in the tissues. A substance would consequently have the greater anæsthetic effect the more it exhausted the oxygen of the tissues. In the condition of anæsthesia the nerve cells possibly become saturated with the anæsthetic, which would fix the oxygen they contain, causing in some measure their asphyxia. In criticising this hypothesis Dr. Huebner points out that if the degree of anæsthetic action of a substance depended upon its oxidation power, it is not explainable why alcohol is a mild anæsthetic and chloral hydrate a powerful one.

### Treatment of Malignant Tumours.

At a recent meeting of the Medical Society in Berlin, Dr. Reicher reported on the results he had obtained in the treatment of malignant tumours in rats and mice by injections of adrenalin. The injections were made in the periphery of the tumours instead of into the tumours themselves. In rats with sarcomatous growths he injected 0.01 centigramme to 0.015 milligramme two or three times a week, and the duration of the treatment was a month or less. This length of treatment was always sufficient to make the tumours disappear or reduce them to the size of a bean. In the latter case the remainder of neoplastic tissue was found to consist of necrotic areas surrounded by a limited zone of inflammatory tissue. The same results were obtained for cancer in mice, but the dose employed need only be a tenth of that used for the rats. Recurrences occurred twice, and then yielded to a repetition of the same treatment. The animals were not otherwise affected by the treatment, and the author has several who have survived four and a half months. He finds that the injections have also a prophylactic action. Thus if injections are made on the day following an inoculation with cancer, the cancer growth only develops in 50 per cent. of the cases instead of in 90 per cent. Moreover, when the tumours do develop, in spite of these injections, they are much smaller than in the control cases. On the other hand, animals cured by injections are not immunised against further inoculations. So far the method does not appear to have been tried on the human subject, and it is impossible to judge from these experiments what therapeutic value it may possess.

### Sterilising the Skin with Petrol and Benzine.

A good deal has been said recently in favour of sterilising the skin of the patient previous to operation by means of tincture of iodine. Dr. Zatti now recommends lavage with petrol and benzine. The region to be operated upon is shaved on the previous day, but no washing or antiseptic compresses are necessary. When the patient is on the operating-table, sterilised towels are placed around in the usual way, care being taken to cover over the mackintosh sheets so as to protect them from the action of the petrol. A large swab of cottonwool soaked in petrol is rubbed well over the skin for a minute or so, and then the skin is similarly treated with a second swab soaked in benzine. The operation is then proceeded with. According to the author this method has the advantage over the application of tincture of iodine that it does not discolour, harden, or irritate the skin; but, on the contrary, makes it supple, smooth, and somewhat unctuous, and at the same time he finds it thoroughly successful in point of sterilisation. He has used the method for the last three years and for more than 700 operations of all varieties. Among others these operations have included 193 radical cures for hernia, 54 laparotomies, seven resections of joints, nine excisions of the breast for cancer, and four operations for goitre. The method has the further advantages of rapidity and simplicity, and the patient is not submitted to the chilling effect of extensive washing.

### Phlegmasia alba dolens.

Dr. Delestre has made a special study of the variations in temperature between the healthy and affected limbs in cases of *phlegmasia alba dolens*. He finds that even in normal conditions the two lower limbs rarely show identically the same temperature, but the differences only amount to a few decimal points, and never reach 1° C., and, again, if the temperature is taken daily for several days variations occur, which, however, also never exceed 1° C. Any difference, therefore, exceeding 1° C. may be taken as abnormal. In cases of phlebitis (the author's observations extend over nine cases) the affected limb shows a rise of temperature of at least 1° C. above that of the sound limb, and the difference may amount to 4° or more. This difference of temperature is at its maximum during the first days of the malady. It generally persists for 25 to 45 days, and may last longer. At the end of this time the temperature curves of the two limbs tend to approximate, and usually the temperature curve of the sound limb is raised a little, so as to meet the descending curve of the affected limb. During the first few days the temperature of the latter varies between 36° and 37° C., and may reach 38.6°. There is no apparent relation between the degree of temperature and the extent of the œdema and pains, nor can one deduce from the temperature chart any indications as to when the patient may get up. But, according to the author, this thermometry may be used as a means of diagnosis in doubtful cases, as, in his opinion, phlebitis may be excluded in any case in which the temperature of the painful limb does not show a rise of at least 1° C. above that of its fellow.

### Chondroma of the Mammary Gland.

Davidsohn, in the *Zentralblatt für Gynäkologie*, reports the case of a woman, aged fifty-one, from whom the right breast was removed for cancer and at the same time from the left breast a tough circumscribed growth the size of a cherry, which shelled out easily. On cutting sections of this and staining them with Kresyl-violet, the growth, under the microscope, proved to be a true enchondroma with characteristic cells and a stroma of pure chondrin. No cancer cells, glandular elements, nor connective tissue were present. According to Virchow cartilaginous tumours, both single and multiple, are common in the mammæ of the dog, but he agrees with Birch-Hirschfield and Ribbert that they are uncommon in the human mammary gland. The last observer considers that chondroma is always part of a mixed growth. Davidsohn points out that on embryological grounds it is not surprising that cartilaginous new growths are uncommon in the mammary gland, and advises extreme care in the staining of sections of growths, especially when of doubtful appearance. In this case chondrin was detected which, but for these precautions having been taken, might easily have escaped notice.

### Experiments in Parthenogenesis.

Some time ago Professor Delage, of the laboratory of Roscoff, reported that he had been able to obtain the parthenogenesis or artificial fecundation of sea-urchins' eggs. He succeeded in keeping alive for a certain time young sea-urchins obtained from eggs which had not been impregnated by the male. This curious phenomenon had been realised by two processes, in the first place by the chemical action of an acid, and in the second by the physical action of an electric current. At a recent meeting of the Paris Academy of Science, M. Bataillon, dean of the Dijon Faculty of Science, reported that he had been able to induce the appearance of a similar phenomenon by purely mechanical means. Taking frog's spawn, each egg was pricked by a platinum wire five-hundredths of a millimetre in diameter, whereupon it commenced to go through the ordinary processes of maturation, precisely as if impregnated naturally, and after the ordinary interval of time burst to give birth to perfectly formed tadpoles, two of which were still living at the time of writing. The author explains this result by the consideration of the phenomena which follow the entrance of the spermatozoon into the egg. In order to produce these, it is merely necessary to produce the first of them, whereupon the others will follow in their natural sequence. The prick of the platinum wire produces an effect analogous to that which follows the penetrating action of the sperm-cell. The author, however, takes care to make it clear that his explanation is merely hypothetical.

### Tenotomy in Fracture Cases.

Mr. Thelwall Thomas, in the *Liverpool Medico-Chirurgical Journal*, pleads earnestly for the more general adoption of tenotomy of the tendo Achillis in certain fractures of the lower limb. First of all he very rightly lays stress upon the importance functionally of obtaining complete correction of deformity in all fracture cases. A very slightly deformed tibia may be many months before its possessor can put his full weight on it and do his full work. He specifies four particular fractures in which the avoidance of deformity is especially difficult: through the lower end of the femur, oblique fracture of the tibia, fracture of the tibia and fibula low down, and fracture of the os calcis. In the least troublesome of these cases success often attends Macintyre's and Roughton's splints, double inclined planes, and stirrup extensions; but in severer cases all these often fail, and for these the alternatives are wiring, screwing, and tenotomy. After an experience of fifteen cases the author speaks very highly of this method of throwing the gastrocnemius out of action, and thus preventing it from pulling on the fragments. The method is a very old one, and he is anxious to rescue it from undeserved neglect and oblivion.

### Proptosis of the Left Broad Ligament.

In a paper published in the *Chicago Medical Recorder*, Mr. W. E. Miles describes in detail the pathology, symptoms, and treatment of proptosis of the left broad ligament as a cause of chronic constipation associated with abdominal pain in women. He claims that in a series of 150 cases he has found the inlet of the pelvic cavity obstructed owing to the assumption by the left broad ligament of a horizontal instead of a vertical position; a shelf is thus formed upon which the loaded sigmoid lies, to which it not uncommonly contracts adhesions, and whose edge frequently kinks the bowel as it curls over into the cavity of the pelvis. In these cases of proptosis the infundibulo-pelvic ligament, instead of arising at the pelvic brim over the left sacroiliac synchondrosis, is found to be attached posteriorly about an inch or an inch and a half below its normal level and nearer to the middle line. The horizontal position of the broad ligament allows the ovary and tube to prolapse into Douglas's pouch, where they often become adherent. The weight of the loaded colon pressing on the shelf causes retroversion of the uterus, and the traction of the lower border of the broad ligament pulls the cervix into the left posterior quadrant of the pelvis. These pathological conditions are associated in many cases with general gastroptosis and enteroptosis, with which they are homologous.

The symptoms which Mr. Miles holds to indicate the obstructive effect of a proptosed broad ligament upon the passage of fæces along the pelvic colon are constipation, left iliac pain, right iliac pain, flatulent distension of the abdomen, gastric disturbances, passage of mucus per rectum, pain in the lumbo-sacral region, and chronic invalidism.

Constipation is constantly found, and in 40 per cent. of cases is first noticed following delivery in a primipara; hence pregnancy is to be regarded as a factor in proptosis of the broad ligament. Though a daily action can be secured by aperient or enema, the quantity voided is insufficient, and the sense of incomplete relief involves fruitless straining; this is owing to the colon being converted into a sort of fæcal reservoir. The characteristic of the left iliac pain, which is the next most important symptom, is that it is not felt during recumbency, but comes on an hour or two after arising in the morning, and gets gradually worse all day; it is often worse during menstruation. Meteorism is common; right iliac pain and mucus passed per rectum are met with only occasionally. To relieve these symptoms the author obliterates the left broad ligament, a proceeding which has cured 148 out of his 150 cases. The patient is placed in the Trendelenburg position and a median incision from umbilicus to pubes is made. Adhesions when present are freed; but if they involve only the posterior third of the surface of the broad ligament they may be left alone. The ovarian vessels are then secured by ligaturing the infundibulo-pelvic ligament close to its origin. The ovary and tube, if diseased, are removed; if not, another ligature is tied round the vessels just clear of the adnexa, and the portion of the free border of the broad ligament between the two ligatures forms the base of the wedge-shaped piece to be removed. The two cut edges of the ligament are then separated, stretched digitally, and carefully united with a continuous suture. Thus all that remains of the broad ligament is a suture line in the peritoneum which lines the lateral wall of the pelvis.

### Lumbar Anæsthesia at First Hand.

In the *Journal of the Royal Army Medical Corps* a captain in that regiment describes his own personal experiences when operated upon for hernia under spinal anæsthesia. He was agreeably surprised with the painlessness of the injection itself, after which he felt no unusual sensation for about two minutes. Then a warm glow spread down the right leg, accompanied by a feeling as if the limb was swelling from increased flow of blood to it. Sensation was lost before motor power, as he could still move the toes after pin-pricks could no longer be appreciated. Anæsthesia was absolute in about six minutes, and the patient was unable to tell when the operation was begun. Still, the skin incision caused a slight feeling of pressure in the region of the left inguinal canal (the operation was on a right inguinal hernia). There was a slight consciousness that the cord was being touched when it was actually manipulated and dragged into the wound. When asked to cough the author did so, and felt as if his abdominal contents were being forced down on to a solid mass in the pelvis. Another very odd sensation was caused by the stitching of Poupart's ligament; the surface of the skin over the splenic region felt as if it were being pressed on by a warm body, and the patient had to satisfy himself by actual palpation before he could disbelieve his senses. The upper limit of anæsthesia was quite abrupt. During the operation no pain was felt, nor any constitutional effect; but afterwards there was slight collapse and nausea for five minutes. As sensation returned there was intense pain in the wound which lasted for six hours and was not controlled by a small dose of morphia; and there was sleeplessness for three nights following.

### Early Stages of General Paralysis.

It is said that much can be accomplished in the treatment of the initial stages of general paralysis, by the injection of solutions of 2 per cent. sodium nucleinate and 2 per cent. sodium chloride. Subcutaneous injections of this solution in different parts of the body are administered in doses of 50 to 100 c.c. at intervals of from five to seven days. These are usually followed by a decided reaction, the temperature rising at times as high as 105° F. and the leucocyte count going up to 61,000. The object of this treatment is to increase oxidation, thereby removing waste-products formed in the disease. The best results are obtained in cases where anti-syphilitic treatment has failed to improve the condition, though Donath, in the *Wiener Klinische Wochenschrifte*, says he has seen good results follow these injections in cases where mercurial treatment was either lacking or insufficiently applied. Among the good effects which follow the treatment by injections are the disappearance of tremor and the periods of excitation, as well as improvements in speech and memory, and the ability to count. In twenty-one of the author's cases, ten were able to resume their occupations, five showed slight improvement, and six were in no way influenced. It is as yet too early to say definitely whether this treatment is capable of effecting a complete cure.

### Prematurely Evacuated Amniotic Fluid.

Schallehn, in the *Arch. f. Gynäkologie*, describes a method whereby, in cases where a premature evacuation of the amniotic fluid causes danger to the fœtus, the fluid can be replaced by the injection of sterile physiological salt solution. This is introduced by means of a rubber balloon provided with a soft rubber tube through which sterilised salt solution can be made to flow. From 500 to 750 c.c. of the fluid are introduced. The introduction of the fluid will bring about regularity and increased force of the uterine contractions. Its use is indicated when, as the result of pressure directly on to the child's circulation by the uterine wall after evacuation of the amniotic fluid, the action of the child's heart becomes weak, and immediate delivery is impossible. It is useful also in cases of muscular atony of the uterus consequent on twin pregnancy, hydramnios or other severe stretching. Premature separation of the placenta can be obviated by its employment, for the pressure is thus equalised and the placental tissue kept in contact with the walls of the uterus. The author reports six cases in which it was successfully used, three in which there was irregularity of the fœtal heart-beat, and three in which, on account of great fœtal mobility, it was desirable to increase the amount of fluid in the uterus.

